# Breaking Up Sedentary Time Reduces Recurrent Fall Risk, but Not Incident Fracture Risk in Older Men

**DOI:** 10.1002/jbm4.10803

**Published:** 2023-08-07

**Authors:** Lauren S. Roe, Stephanie Harrison, Peggy M. Cawthon, Kristine Ensrud, Kelley Pettee Gabriel, Deborah M. Kado, Jane A. Cauley, the Osteoporotic Fractures in Men (MrOS) Study Research Group

**Affiliations:** ^1^ Department of Epidemiology University of Pittsburgh School of Public Health Pittsburgh PA USA; ^2^ California Pacific Medical Center Research Institute San Francisco CA USA; ^3^ Department of Epidemiology and Biostatistics University of California, San Francisco San Francisco CA USA; ^4^ Division of Epidemiology and Community Health, Department of Medicine University of Minnesota Minneapolis MN USA; ^5^ Center for Care Delivery and Outcomes Research Minneapolis VA Health Care System Minneapolis MN USA; ^6^ Department of Epidemiology The University of Alabama at Birmingham Birmingham AL USA; ^7^ Stanford University Department of Medicine Palo Alto CA USA; ^8^ Geriatric Research Education and Clinical Center (GRECC) Veterans Administration Health System Palo Alto CA USA

**Keywords:** AGING, EPIDEMIOLOGY, EXERCISE, FALLS, FRACTURES, PHYSICAL ACTIVITY

## Abstract

Apart from physical activity volume, frequent breaks from sedentary bouts and active bouts may differentially reduce fall and fracture risk. We assessed the longitudinal relationship between frequency of breaks from time spent sedentary and frequency of active bouts with recurrent falls and fractures. The sample included 2918 men aged 79.0 ± 5.1 years with free‐living activity (SenseWear Armband) at the Osteoporotic Fractures in Men Study (MrOS) year 7 (2007–2009) visit. Men were divided into quartiles by the number of breaks from sedentary bouts (sedentary bout: 5+ minutes sedentary; <1.5 metabolic equivalents of task [METS]) and separately by active bout frequency (active bout: 5+ minutes of activity; ≥1.5 METS). Recurrent falls (2+ falls/year) and fractures were ascertained by self‐report; fractures were radiographically confirmed. Generalized estimating equations estimated the recurrent fall odds, with restricted cubic splines applied to assess nonlinear relationships. Cox proportional hazards models estimated fracture risk. Over 4 years of follow‐up after year 7, 1025 (35.1%) men were fallers. Over 8.40 ± 4.10 years of follow‐up, 640 (21.9%) men experienced a fracture. There was a significant nonlinear U‐shaped relationship between number of breaks from sedentary bouts and recurrent falls (*p* < 0.001); compared with men with few breaks from sedentary bouts (1.4–<13.6), the odds of recurrent falls were lower with a moderate number (13.6–<17.0, odds ratio [OR] = 0.82, 95% confidence interval [CI] 0.66, 1.01; 17.0–<20.4, OR = 0.79, 95% CI 0.64, 0.99), but not with the highest number of breaks from sedentary bouts (20.4–34.6, OR = 1.01, 95% CI 0.81, 1.27). Results remained borderline significant after adjusting for total sedentary time. Men with the highest compared with the lowest number of breaks from sedentary bouts had a lower fracture risk, but the association was attenuated after adjustment for total sedentary time. No associations were observed for active bout frequency. In conclusion, breaking up extended periods of sedentary time reduces fall risk regardless of total sedentary time. © 2023 The Authors. *JBMR Plus* published by Wiley Periodicals LLC. on behalf of American Society for Bone and Mineral Research.

## Introduction

Falls and subsequent fractures are common among the growing population of older adults aged 65 years and older, resulting in an estimated annual cost of $50 billion.^(^ ^1^, ^2^ ^)^ In randomized controlled trials, increasing exercise has successfully reduced fall rate^(^ ^3^ ^)^ and fall‐related injury.^(^ ^4^ ^)^ Although no sufficiently powered randomized trial exists looking at incident fractures, observational studies report lower fracture risk with more aerobic exercise or resistance training.^(^ ^5^ ^)^ Importantly, lack of long‐term adherence to exercise programs may reduce observed benefits from structured exercise, so real‐world approaches to improve bone health need to be explored.^(^ ^5^ ^)^

Accelerometer‐based devices reliably and validly^(^ ^6^ ^)^ measure body position^(^ ^6^ ^)^ and the amount and intensity of daily physical activity continuously throughout a 24‐hour day. Previous studies assessing the relationship between falls and accelerometer‐measured total daily physical activity report mixed results,^(^ ^7^, ^8^, ^9^, ^10^, ^11^, ^12^ ^)^ with differences attributed to mobility,^(^ ^8^ ^)^ functional status,^(^ ^9^ ^)^ and age^(^ ^7^, ^9^ ^)^ of the study populations. Fracture risk may also be lowered by 11% to 40% with higher amounts of physical activity.^(^ ^5^ ^)^ Many of these studies report on the average number of minutes per day in different intensities of activity, but the way in which each of these activities are accumulated may also be an important exposure.^(^ ^5^ ^)^

Sedentary bouts are defined as prolonged periods of sedentary behavior.^(^ ^13^ ^)^ More frequently taking a break from sedentary bouts and inherently ending the sedentary activity may provide biologic benefit^(^ ^14^, ^15^, ^16^, ^17^ ^)^ to muscle strength^(^ ^5^ ^)^ and muscle power^(^ ^18^ ^)^ in some older adults by acting as a sit‐to‐stand exercise throughout the day and also preparing someone to become active.^(^ ^14^, ^15^, ^16^, ^17^ ^)^ These adaptations may reduce fall and fracture risks.^(^ ^19^ ^)^ Similarly, someone with more prolonged (5+ minutes) periods of activity at any intensity may have lower fall and fracture risks because they are able to sustain activity. Thus, we aimed to determine the association between the number of breaks in prolonged sedentary time (eg, breaks from sedentary bouts) and the frequency of active bouts (5+ minutes active) with falls and fractures. We hypothesized that a higher number of breaks from sedentary bouts and more frequent active bouts would be separately associated with a lower risk of recurrent falls (≥2 falls per year) and with lower risk of any incident clinical fracture.

## Materials and Methods

### Study design

To address our hypothesis, we conducted a longitudinal analysis in 2918 men participating in the year 7 (2007–2009) study visit of the Osteoporotic Fractures in Men (MrOS) Study. MrOS is an ongoing longitudinal cohort study that enrolled 5994 men aged 65 years and older at baseline (2000–2002) from six clinical centers in the United States (Birmingham, AL; Minneapolis, MN; Palo Alto, CA; Monongahela Valley near Pittsburgh, PA; Portland, OR; and San Diego, CA).^(^ ^20^, ^21^ ^)^ Men able to walk without assistance and without a history of a bilateral hip replacement were eligible to participate. Among the 3354 men with available activity monitor data from the year 7 visit, 2918 men had valid data, defined as wearing the accelerometer for five or more 24‐hour periods with at least 90% wear time.^(^ ^22^ ^)^ Each study site's institutional review board approved the study protocol, and all participants provided written and informed consent before participating.

### Accelerometry

Immediately after the year 7 visit, participants wore the SenseWear Pro3 Armband by Body Media Inc. (Pittsburgh, PA, USA) on their right arm continuously for 7 days, except when showering or participating in water activities. Data were collected in 1‐minute epochs using a combination of six sensors. Metabolic equivalents of task (METs) and time spent lying down were calculated from proprietary algorithms from Innerview Professional 5.1 software developed by Body Media Inc. using raw accelerometry data and participant data (height, weight, age, handedness, and smoking status). Average METs were estimated from the algorithm using predicted energy expenditure calculations for resting metabolic rates,^(^ ^23^ ^)^ which has been validated against doubly labeled water in older adults^(^ ^24^ ^)^ and an accurate measure of energy expenditure for ambulation.^(^ ^25^ ^)^ The predicted MET for each minute was then characterized as sedentary (<1.5 METs)^(^ ^26^ ^)^ or active (≥1.5 METs).

Average number of breaks from prolonged time spent sedentary (eg, number of breaks from sedentary bouts) and active bout frequency was determined from the year 7 accelerometry data. A sedentary bout was defined as 5+ minutes in continuous sedentary behavior. The number of breaks from sedentary bouts was determined by the number of times the participant transitioned to activity of any intensity for any amount of time after a sedentary bout.^(^ ^13^ ^)^ Sleep was excluded entirely from this analysis. More breaks from sedentary bouts equated to a more active individual, whereas fewer breaks equated to a more sedentary individual with longer periods of continuous sedentary behavior. The number of active bouts was defined as at least five continuous minutes in at least light activity. Less frequent bouts of activity equated to a less active individual because that individual did not complete many (if any) bouts of continuous activity lasting at least 5 minutes.

Although number of breaks from sedentary bouts and number of active bouts were highly correlated (Spearman correlation coefficient = 0.74, *p* < 0.001), the break from the sedentary bout does not necessarily equate to an active bout; the time in activity at any intensity after the sedentary bout could last fewer than 5 minutes, whereas an active bout is defined here as 5+ minutes in activity of any intensity. Similarly, zero active bouts does not necessarily mean continuous sedentary behavior but rather activity that never reached the 5+‐minute threshold used for this study (Supplemental Fig. S1).

Both the number of breaks from sedentary bouts and number of active bouts were divided into quartiles to determine the shape of the association. The number of breaks from sedentary bouts was 1.4–<13.6, 13.6–<17.0, 17.0–<20.4, and 20.4–34.6 for Q1–Q4. The number of active bouts was 0–<5.0, 5.0–<7.6, 7.6–<10.8, and 10.8–26.4 for Q1–Q4. For both behaviors, the first quartile (Q1) formed the referent group.

### Recurrent falls and fractures

Recurrent falls were assessed over 4 years of follow‐up, after the year 7 visit. Self‐reported falls were obtained through triannual mailed questionnaires. Falls (yes/no) were ascertained by asking, “Have you fallen in the past 4 months?” In the current analysis, recurrent falls were defined as at least two falls in 1 year. The sample size for recurrent falls was 2910, as 8 participants were either terminated or withdrawn during the first year of follow‐up and did not have enough data to categorize as a recurrent faller or not.

Incident clinical fractures were also reported on the triannual questionnaire and were centrally confirmed through physician review of radiographic reports. Fractures from severe trauma were included because these fractures have been linked to low bone mineral density (BMD).^(^ ^27^ ^)^ Follow‐up time was determined by date of incident fracture, last postcard questionnaire contact, most recent visit date, or if the participant died or was terminated. Postcard follow‐up was greater than 95% complete among active surviving participants.

### Potential confounding variables

Race, education, and marital status were collected at study baseline (2000–2002). All other potential confounding variables were collected during the year 7 visit, the same visit as accelerometer assessment. Self‐reported age, current smoking status, and number of physician‐diagnosed selected medical conditions (range 0–9) were collected during the in‐person interview. Height was measured using a wall‐mounted stadiometer, and weight on a balance beam or digital scale. Height and weight were used to calculate body mass index (BMI) in kilograms per meters squared. Total hip BMD was measured as grams per centimeter squared using dual‐energy X‐ray absorptiometry (DXA; Hologic, Inc., Bedford, MA, USA).^(^ ^28^ ^)^ A questionnaire was used to obtain information about the men's ability to complete five instrumental activities of daily living (IADL), including ability to manage money, bathe/shower, get in and out of bed/chairs, and manage medications.^(^ ^29^ ^)^ Fall history was obtained by asking participants if they had fallen in the past 12 months. If yes, they were asked if they broke or fractured a bone. Finally, physical performance was measured from chair stand speed, hand‐grip strength, and gait speed. Chair stands were calculated as the number of chair stands completed in 10 seconds. The Jamar dynamometer (Sammons Preston Rolyan, Bolingbrook, IL, USA) was used to measure grip strength, and gait speed at usual pace was measured during a 6‐meter walk test expressed as m/s.

### Statistical approach

Descriptive statistics were generated for participant characteristics, exposure variables, and confounding variables overall and by both recurrent fall status and incident clinical fracture status. Continuous variables are presented as means and standard deviations or median (quartile 1, quartile 3), and categorical variables were presented as count and percentages. Normality of continuous variables was assessed using the Shapiro–Wilk test for normality. All continuous variables were skewed except for height, so differences by fall status and fracture status were tested using Wilcoxon tests for continuous variables. A chi‐square test was used to test differences in categorical variables.

A generalized estimating equation (GEE) using a binomial distribution with an independent correlation structure (based on QIC) was used to model the association between repeated measures of annually self‐reported recurrent falls with number of breaks from sedentary bouts and number of active bouts separately. Linearity of the relationship was assessed nonparametrically with restricted cubic splines when breaks from sedentary bouts and active bouts were entered into the models as continuous variables (versus quartiles). The tests for nonlinearity used the likelihood ratio test to compare the models with only a linear term to a model with a linear and a cubic term (ie, test for curvature)^(^ ^30^ ^)^ in unadjusted and fully adjusted models.

The base model (model 1) included age, race, clinic, and season when activity monitor was worn. Model 2 included the following additional covariates: height, weight (as done previously),^(^ ^22^ ^)^ self‐reported history of diabetes, health status, smoking, number of chronic health conditions, and number of IADL impairments. Finally, physical performance measures were added into the model one at a time^(^ ^5^, ^15^, ^16^ ^)^ to determine if physical performance impacted any observed associations. A 10% change in the effect estimates suggested attenuation.^(^ ^31^ ^)^

Kaplan–Meier curves were used to describe the cumulative incidence of fractures by number of breaks from sedentary bouts and number of active bout quartile. Cox proportional hazards regression were used to evaluate incident fractures. Models were adjusted for the same base model covariates and model two covariates described for the GEE models, with the addition of total hip BMD and fall and fracture history. Finally, a sensitivity analysis was conducted to determine if mortality was a competing risk for incident fractures using the Fine and Gray method.^(^ ^32^ ^)^ SAS 9.4 (SAS Institute, Inc., Cary, NC, USA) was used to conduct this analysis.

## Results

This analysis included 2918 men aged 79.03 ± 5.15 years at the MrOS year 7 visit (2007–2009; analytic baseline). Overall, men were mostly white (91.7%), highly educated with a college degree or more (79.3%), and mostly in good health (87.2%) with few medical conditions (0–1: 55.5%; Table 1). The median number of days the accelerometer was worn was 5 days. Over 4 years of follow‐up, 1025 (35%) men were characterized as recurrent fallers (2+ falls/year). Over 8.40 ± 4.10 years, 640 (21.9%) men experienced an incident clinical fracture.

**Table 1:** Overall Sample Characteristics at the Year 7 Visit for the 2918 MrOS Men Included in This Analysis

Characteristic	Overall
Age (years)	78 (75, 83)
White, %	2676 (91.7)
Average wear time (days)	5 (5, 5)
College degree or higher	2315 (79.3)
Married	2289 (78.4)
Chronic health conditions	
0–1 conditions	1618 (55.5)
2–3 conditions	1180 (40.5)
≥4 conditions	118 (4.1)
Diabetes history	435 (14.9)
Self‐reported good/excellent health	2543 (87.2)
Current smoker	53 (1.8)
Average time spent in activities of the following intensities (min/d)	
Sedentary (<1.5 METS)	844.7 (772.2, 915.0)
Light (1.5–<3.0 METS)	64.0 (42.4, 88.4)
Moderate (3.0–<6.0 METS)	65.0 (37.0, 104.0)
Vigorous (6.0 + METS)	4.2 (1.2, 9.8)
Any intensity (≥1.5 METS)	134.1 (83.8, 201.8)
PASE score	132.8 (87.9, 176.2)
Average sedentary bout frequency	17.0 (13.6, 20.4)
Average active bout frequency	7.6 (5.0, 10.8)
Height (cm)	173.39 ± 6.84
Weight (kg)	80.3 (72.2, 89.0)
BMI (kg/m^2^)	26.7 (24.5, 29.2)
Total hip BMD (g/cm^2^)	0.94 (0.85, 1.04)
IADL impairment (≥1)	634 (21.75)
Mini‐mental state (0–100)	94 (90, 97)
Grip strength, kg	38 (34, 44)
Gait speed (m/s)	1.15 (0.99, 1.29)
Chair stand per 10 s	4.4 (3.6, 5.3)

*Note*: Continuous variables presented as median (quartile 1, quartile 3) or mean ± SD, and categorical variables presented as *n* (%).

Abbreviation: BMD = bone mineral density; BMI = body mass index; IADL = instrumental activity of daily living; METS = metabolic equivalents; MrOS = Osteoporotic Fractures in Men Study; PASE = physical activity scale for the elderly.

At the year 7 clinic visit, men who reported recurrent falls over the 4 years of follow‐up were more likely to be older, not married, less active, to have more medical conditions, and to have poorer health and measured physical performance compared with men who were not recurrent fallers (Supplemental Table S1, all *p* < 0.001). Men who fell were more likely to be sedentary and more likely to spend fewer minutes in total activity, but total time awake was similar between recurrent fallers and men who did not fall (16.6 ± 1.4 hours versus 16.6 ± 1.5 hours, *p* = 0.95). Similarly, recurrent fallers were more likely to have an average of one fewer breaks from 5+ minutes sedentary and one fewer active bouts per day than men who did not fall (*p* < 0.001).

Men who experienced a fracture tended to be older, more highly educated, and with a lower BMI and lower hip BMD (Supplemental Table S1, all *p* < 0.001). Men who had an incident clinical fracture spent the same amount of time awake (16.5 ± 1.4 hours versus ±1.5 hours, *p* = 0.11) as did men who did not have a clinical fracture. There was no significant difference in sedentary time, active time, number of breaks in time spent sedentary, or active bout frequency among those who experienced a subsequent fracture versus those who did not.

### Sedentary and active bouts and recurrent falls

**Fig. 1:**
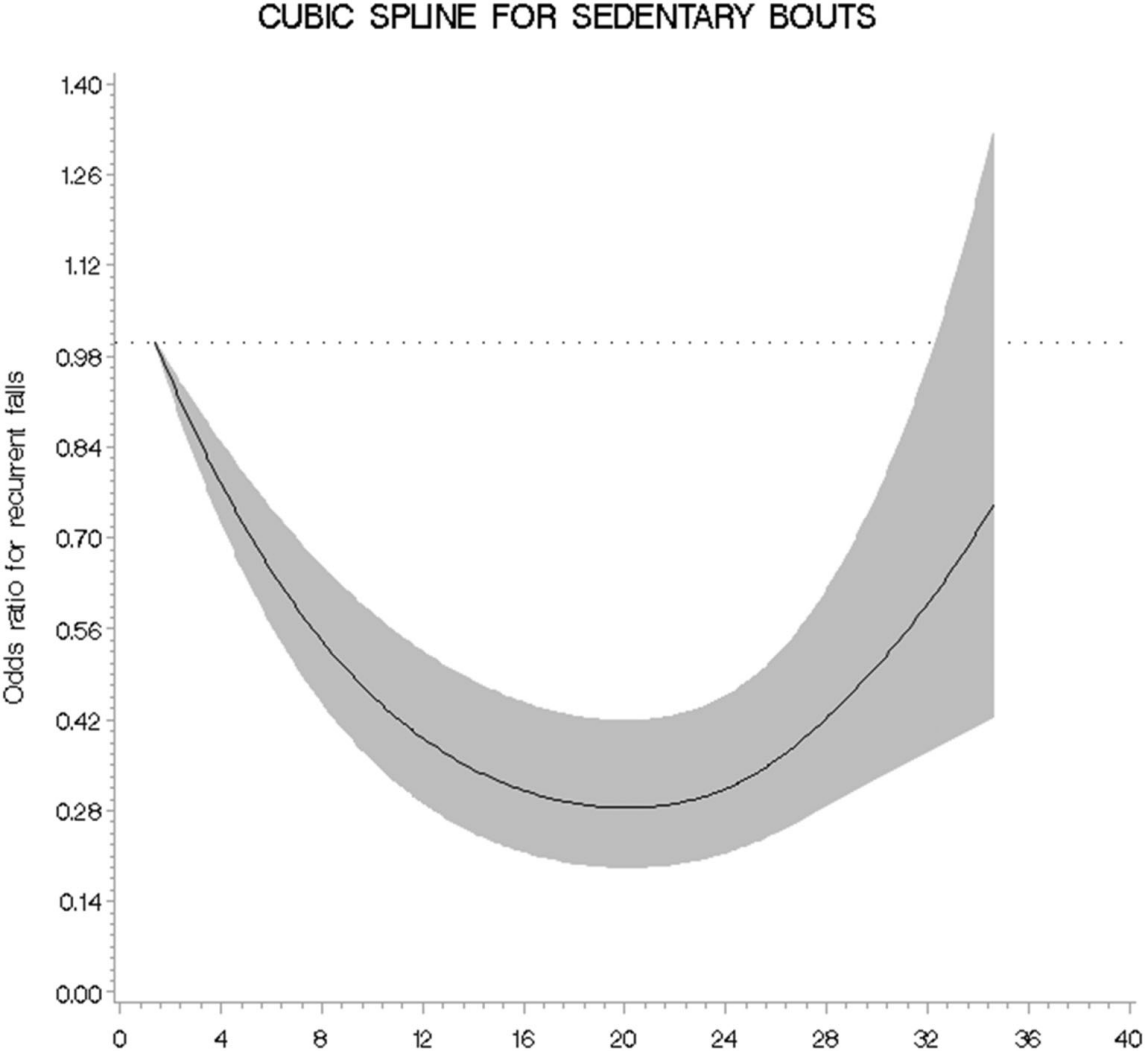
Cubic spline graph for the odds of recurrent fall by frequency of breaks from a sedentary bout after adjustment for model 2 variables. Model 2: Age, race, clinic, season when accelerometer was worn, height, weight, self‐reported history of diabetes, health status, smoking, number of comorbidities, instrumental activities of daily living impairments, total hip bone mineral density, fracture history, and fall history.

After adjustment for age, race, clinic, and season of accelerometer wear (model 1), men with a moderate number of breaks from sedentary bouts (13.6–<20.4 breaks, Q2 and Q3) were less likely to have recurrent falls (Table 2). However, men with the highest number of breaks from sedentary bouts (20.4–<34.6 breaks, Q4) had a higher likelihood of recurrent falls; the likelihood ratio test with restricted cubic splines suggested a significant nonlinear U‐shaped association between the number of breaks from sedentary bouts as a continuous variable with recurrent falls (Fig. 1). Subsequent adjustment for potential confounding variables in model 2 (model 1 + height, weight, self‐reported history of diabetes, health status, smoking, number of comorbidities, IADL impairments) attenuated some of the observed relationships from model 1. Men with 20.4–<34.6 (Q4) breaks from sedentary bouts did not have a significantly different risk in recurrent falls risk compared with men with 1.4–<13.6 (Q1) breaks from sedentary bouts. However, men with 13.6–<17.0 (Q2) and 17.0–<20.4 (Q3) breaks from sedentary bouts had a significant or borderline significant reduction in recurrent fall risk compared with men with 1.4–<13.6 (Q1) breaks from sedentary bouts (Table 2). The test for curvature using the restricted cubic splines reflected these results by indicating a significant nonlinear relationship between the number of breaks from sedentary bouts as a continuous variable with recurrent falls (*p* < 0.001).

**Table 2:** Generalized Estimating Equations for Estimates of Odds of Experiencing Recurring Falls by Quartiles of Breaks From Sedentary Bouts and Active Bout Frequencies

Odds ratio (95% confidence interval)			
	Model 1^a^	Model 2^b^	Model 2 + total sedentary or active time^c^
Frequency of breaks from sedentary bouts			
Q1 (1.4–<13.6 breaks from sedentary bouts)	1.00 (Referent)	1.00 (Referent)	1.00 (Referent)
Q2 (13.6–<17.0 breaks from sedentary bouts)	0.67 (0.55, 0.82)	0.82 (0.66, 1.01)	0.83 (0.67, 1.03)
Q3 (17.0–<20.4 breaks from sedentary bouts)	0.60 (0.49, 0.75)	0.79 (0.64, 0.99)	0.81 (0.65, 1.01)
Q4 (20.4–34.6 breaks from sedentary bouts)	0.74 (0.60, 0.92)	1.01 (0.81, 1.27)	1.04 (0.83, 1.30)
*p* value^d^	<0.001	<0.001	<0.001
Frequency of active bouts per day			
Q1 (0–<5.0 bouts)	1.00 (Referent)	1.00 (Referent)	1.00 (Referent)
Q2 (5.0–<7.6 bouts)	0.74 (0.60, 0.91)	0.90 (0.73, 1.11)	0.91 (0.73, 1.14)
Q3 .6–<10.8 bouts)	0.71 (0.58, 0.88)	0.93 (0.75, 1.15)	0.95 (0.73, 1.24)
Q4 (10.8–26.4 bouts)	0.74 (0.60, 0.91)	1.01 (0.80, 1.26)	1.06 (0.72, 1.56)
*p* value^d^	0.004	0.74	0.53

*Note*: Frequency of breaks from sedentary bouts was defined as the number of times per day each participant took a break from a sedentary bout that lasted 5 minutes or more, excluding sleep. Frequency of active bouts was defined as the number of times per day each participant had uninterrupted active time lasting 5 minutes or more.

^a^ Model 1: age, race, clinic, season when accelerometer was worn.

^b^ Model 2: model 1 + height, weight, self‐reported history of diabetes, health status, smoking, number of comorbidities, instrumental activities of daily living impairments.

^c^ Frequency of sedentary bouts per day model adjusted for total sedentary time. Frequency of active bouts per day adjusted for total active time.

^d^ Likelihood ratio test in a nonparametric cubic spline to test the linear term of the number of sedentary or active bouts as a continuous variable with the cubic spline term (ie, test for curvature).

Further adjustment for physical function measures that may be potential mechanisms linking bouts with falls slightly attenuated observed results from model 2, but not by ≥10%, the threshold used to determine attenuation (Supplemental Table S2). After adjusting for total time sedentary in the number of breaks from sedentary bouts model, results were slightly attenuated (Table 2), but overall likelihood test remained statistically significant.

After adjustment for age, race, clinic, and season of accelerometer wear, men with more frequent active bouts compared with men with the fewest active bouts (<5.0 bouts, Q1) were significantly less likely to have recurrent falls, similar to that observed for frequency of sedentary bouts (model 1; Table 2, *p* value for nonlinear relationship from likelihood ratio test with restricted cubic splines = 0.004). However, all associations were attenuated and no longer significant after adjusting for variables in model 2. Results were similar when adjusting for physical function measures (Supplemental Table S2) or for total time in activity at any intensity (Table 2).

### Associations of number of breaks from sedentary bouts and frequency of active bouts with incident fracture

**Fig. 2:**
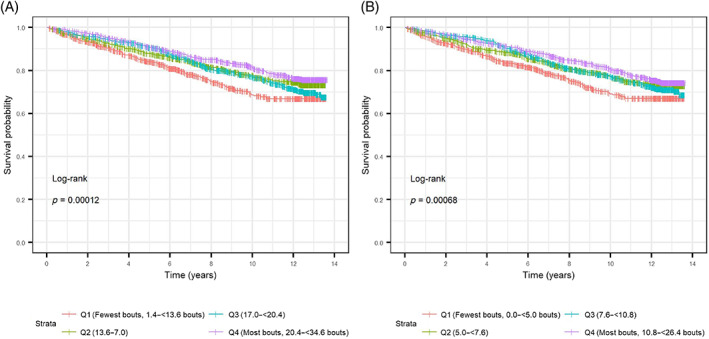
(*A*) Cumulative survival of fractures by quartiles of frequency of breaks from sedentary bouts, excluding sleep. Frequency of breaks from sedentary bouts was defined as the number of times per day each participant took a break from a sedentary bout that lasted 5 minutes or more, excluding sleep. (*B*) Cumulative survival of fractures by quartiles of frequency of active bouts (bouts/day), excluding sleep. Frequency of active bouts was defined as the number of times per day each participant had uninterrupted active time lasting 5 minutes or more.

The probability of surviving without an incident fracture over the average follow‐up time of 8.40 ± 4.10 years was the highest among men with the highest number of breaks from sedentary bouts (Q4) and lowest among men with the lowest number of breaks from sedentary bouts (Q1; log rank *p* < 0.001; Fig. 2*A*). Similarly, the probability of surviving without an incident fracture over the follow‐up period was the highest among men with the highest frequency of active bouts (Q4, most active) and lowest among men with the lowest frequency of active bouts (Q1, least active; log rank *p* < 0.001; Fig. 2*B*).

Cox proportional hazards regression models indicated a significantly lower risk of fractures between men with more breaks from sedentary bouts compared with men with the lowest number of breaks from sedentary bouts (*p*‐trend = 0.04), after adjustment for age, sex, clinic, and season of accelerometer wear (Table 3). However, after adding additional variables in model 2, results were attenuated for men with 13.6–<17.0 breaks from sedentary bouts (Q2) and men with 17.0–<20.4 breaks from sedentary bouts (Q3) versus men with 1.4–<13.6 breaks from sedentary bouts (Q1), and the linear trend was no longer statistically significant. In model 2, men with the highest number of breaks from sedentary bouts (Q4) had a significantly lower risk of incident fracture compared with men with the fewest breaks from sedentary bouts (Q1; hazard ratio [HR] = 0.75, 95% confidence interval [CI] 0.59, 0.94). Results did not change after adding gait speed to the model, but results for Q4 versus Q1 were attenuated when grip strength and chair stands in 10 seconds (Supplemental Table S2, nonlinear relationship *p* = 0.02) were separately added to model 2. Similarly, associations were attenuated and no longer significant when total time sedentary was added to model 2 (Table 3).

**Table 3:** Cox Proportional Hazards Models to Estimate Association of Quartile of Frequency of Breaks From Sedentary Bouts and Active Bouts With Incident Clinical Fractures

Hazard ratio (95% confidence interval)			
	Model 1^a^	Model 2^b^	Model 2 + total sedentary or active time^c^
Frequency of breaks from sedentary bouts			
Q1 (1.4–<13.6 breaks from sedentary bouts)	1.00 (Referent)	1.00 (Referent)	1.00 (Referent)
Q2 (13.6–<17.0 breaks from sedentary bouts)	0.79 (0.63, 0.99)	0.85 (0.67, 1.07)	0.85 (0.68, 1.09)
Q3 (17.0–<20.4 breaks from sedentary bouts)	0.88 (0.71, 1.10)	0.96 (0.76, 1.21)	0.97 (0.77, 1.23)
Q4 (20.4–34.6 breaks from sedentary bouts)	0.75 (0.59, 0.94)	0.77 (0.60, 0.99)	0.79 (0.61, 1.01)
*p*‐trend	0.04	0.11	0.15
Frequency of active bouts per day^d^			
Q1 (0–<5.0 bouts)	1.00 (Referent)	1.00 (Referent)	1.00 (Referent)
Q2 (5.0–<7.6 bouts)	0.76 (0.62, 0.97)	0.86 (0.68, 1.08)	0.80 (0.63, 1.03)
Q3 (7.6–<10.8 bouts)	0.85 (0.68, 1.06)	0.91 (0.72, 1.15)	0.80 (0.60, 1.06)
Q4 (10.8–26.4 bouts)	0.77 (0.61, 0.97)	0.84 (0.66, 1.08)	0.65 (0.43, 0.98)
*p*‐trend	0.07	0.28	0.06

*Note*: Frequency of breaks from sedentary bouts was defined as the number of times per day each participant took a break from a sedentary bout that lasted 5 minutes or more, excluding sleep. Frequency of active bouts was defined as the number of times per day each participant had uninterrupted active time lasting 5 minutes or more.

^a^ Model 1: age, race, clinic, season when accelerometer was worn.

^b^ Model 2: Model 1 + height, weight, self‐reported history of diabetes, health status, smoking, number of comorbidities, instrumental activities of daily living impairments, total hip bone mineral density, fracture history, and fall history.

^c^ Frequency of sedentary bouts per day model adjusted for total sedentary time. Frequency of active bouts per day adjusted for total active time.

^d^ Proportional hazards assumption is violated for all models.

For active bout frequencies, men in Q2 and Q4 had a reduction in risk of incident fracture after adjustment for variables in model 1 (Q2 HR = 0.76, 95% CI 0.62, 0.97; Q4 HR = 0.77, 95% CI 0.61, 0.97). Results were completely attenuated after adding covariates in model 2, physical function measures, and total time in activity at any intensity (data not shown).

### Sensitivity analysis

The association between quartiles of number of breaks from sedentary bouts with the cumulative incidence of both fractures and all‐cause mortality were both significant according to the Fine and Gray model (Supplemental Table S3), indicating death was a competing risk for fractures. For active bout frequencies, the cumulative incidence of all‐cause mortality was significant, but cumulative incidence function was not significant for fractures. This indicates that death was related to active bout frequencies, but incident fractures was not. Models were adjusted for variables in model 2.

## Discussion

Our results suggest that a moderate number of breaks in sedentary bouts (sedentary bouts: 5+ minutes of uninterrupted sedentary time) reduces fall risk regardless of total time spent sedentary. The associations between breaking up sedentary time and fracture risk, and between frequency of active bouts and fractures, were not independent common risk factors for fractures; the observed associations were not significant after adjusting for total time sedentary or active and after adding physical performance measures into the models. These conclusions partially support our hypothesis that a higher number of breaks from sedentary bouts are associated with a lower risk of recurrent falls but only up to a certain point because a significant nonlinear relationship observed. Contrary to our hypothesis, no associations were detected between active bout frequencies and recurrent falls or fractures.

Device‐based sedentary and active bouts examined in the present analysis is a novel exposure for recurrent falls and fractures in older adults. Previous cohort studies that examined objectively measured bouts of activity only looked at the length of the bout,^(^ ^8^, ^11^, ^33^ ^)^ not how often the bouts occurred during the day. Nonetheless, results from the current study align with results of prior studies examining bout length with fall risk. Among older men with mobility limitations (defined as some, moderate, or severe difficulty getting about outdoors), every 30 minutes of sedentary behavior was associated with a 15% higher fall risk,^(^ ^8^ ^)^ but there was no association among men without mobility limitations. We did not assess interactions between the performance variables measured (chair stand, gait speed, and grip strength) with number of breaks from sedentary bouts, so we cannot support or refute those findings. Instead, we assessed whether any of these variables attenuated any of the results. Our results did not differ by the 10%, suggesting results were not attenuated once physical performance measures were added to the models.^(^ ^31^ ^)^ Similarly in older women, a linear association between sedentary bout duration and falls was observed among women with increasingly longer sedentary bouts (eg, less active).^(^ ^11^ ^)^ Although the exposure of bout length and number of breaks are slightly different, the results are consistent with one another in that frequent breaks from sedentary bouts and shorter average sedentary bouts are beneficial for older adults to reduce fall risk.

Previous research examining bouts of activity with fractures is much more limited. Meta‐analyses support exercise as an effective intervention for reducing fall‐related injuries^(^ ^34^ ^)^ and fall‐related fractures,^(^ ^4^ ^)^ but the evidence is limited to total time spent in activities rather than bouts of activity.^(^ ^5^ ^)^ Similar to the current analysis, studies are likely underpowered to detect any differences, particularly when examining quartiles of breaks from sedentary bouts and active bout frequencies. Moreover, the type of activity (eg, weight‐bearing versus non‐weight‐bearing) may be more important than the pattern of accumulation for fracture risk.

The associations observed between breaks from sedentary bouts with falls and fractures is biologically plausible based on previous research. There is strong evidence that functional strength reduces fall risk from randomized clinical trials.^(^ ^34^ ^)^ For some, breaking up sedentary behavior may be the most activity performed in a day and act as a functional strength movement by simulating a sit‐to‐stand movement. Performing this movement when ending a sedentary bout could promote muscular strength^(^ ^5^ ^)^ and also gets a person in a ready position to be physically active. Given there are several other risk factors for falls like balance impairment, for others who are more frail, they may not transition out of sedentary often, so there is less of an opportunity to fall. Meanwhile, men who are up and down a lot (eg, more breaks from sedentary bouts) may have the same fall risk because they may have more opportunity to fall from transitioning up and down, but they are possibly stronger and functionally more stable. This notion is consistent with the U‐shaped relationship.

Previous evidence has also demonstrated that increased activity improves bone strength,^(^ ^14^, ^15^ ^)^ bone geometry,^(^ ^14^ ^)^ muscle strength and power,^(^ ^15^, ^16^ ^)^ and balance.^(^ ^17^ ^)^ This only explains why the observed relationship between breaks from sedentary bouts and fracture risk was attenuated after adjusting for total sedentary time and physical performance, but the null findings between active bout frequencies with falls and fractures do not align with this evidence. The duration of activity time or the type of activity could be more important than active bout frequency in reducing fall and fracture risk, as demonstrated previously in the MrOS cohort.^(^ ^7^ ^)^

Results from this study are strengthened by the large sample size with >90% follow‐up, objective measures of daily activity, confirmed fracture status, and frequent contact with participants to update fall status. However, there were several limitations. These results are not generalizable to other older adult populations, as this sample consists of a homogenous sample of mostly healthy older white men. Falls were not defined in the mailed questionnaires, so participants may underreport falls if they did not include falls where they did not hit the ground, for example. This could bias the results toward the null. It was also not possible to confirm self‐reported falls, obtain information about circumstances leading to the fall, or examine the outcome of injurious falls. Time‐varying analysis may be more informative of the association between daily activities and fall and fracture risk, but this information was not collected for the study. Finally, survival bias is likely to exist for this sample. These men were in their late 70s at baseline of this analysis, so they are likely to represent a healthy and more active sample of older adults than the general population.

In conclusion, results of this study suggest a U‐shaped relationship between number of breaks from sedentary bouts per day and recurring falls, where too few or too many sedentary breaks increases fall risk but moderately frequent breaks from sedentary bouts throughout the day may reduce recurrent fall risk. The least sedentary men had a lower risk of fracture that was partially explained by improved physical performance. However, being active for at least 5 minutes frequently throughout the day did not impact fall risk or fracture risk. There are several possibilities for future research based on these results, including further exploration of the shape of the relationship between sedentary bout frequencies and the timing of when the breaks occur throughout the day.

## Author Contributions

**Jane A. Cauley:** Conceptualization; funding acquisition; investigation; project administration; resources; supervision; validation; writing – original draft; writing – review and editing. **Lauren S. Roe:** Conceptualization; data curation; formal analysis; investigation; methodology; project administration; software; validation; visualization; writing – original draft; writing – review and editing. **Stephanie Harrison:** Conceptualization; data curation; investigation; methodology; project administration; software; validation; writing – review and editing. **Peggy M. Cawthon:** Conceptualization; data curation; funding acquisition; investigation; methodology; project administration; resources; supervision; validation; writing – review and editing. **Kristine Ensrud:** Funding acquisition; investigation; project administration; resources; supervision; validation; writing – review and editing. **Kelley Pettee Gabriel:** Investigation; validation; writing – review and editing. **Deborah Kado:** Funding acquisition; investigation; writing – review and editing.

## Disclosures

The authors declare no conflicts of interest.

### Peer Review

The peer review history for this article is available at https://www.webofscience.com/api/gateway/wos/peer-review/10.1002/jbm4.10803.

## Supporting information


**Data S1.** Supporting Information.

## Data Availability

The data that support the findings of this study are openly available at https://mrosonline.ucsf.edu/. The code that supports the findings are available from the authors upon reasonable request.
