# Supplementary material for: Breaking Up Sedentary Time Reduces Recurrent Fall Risk, but Not Incident Fracture Risk in Older Men
Source: JBMR Plus. 2023 Aug 7;7(12):e10803. doi: 10.1002/jbm4.10803 (PMC10731095; doi:10.1002/jbm4.10803)
Supplement: Supplementary file 1 — Data S1. Supporting Information. [file JBM4-7-e10803-s001.docx]

|  | **Non-fallers (n=1885)*** | **Fallers (n=1025)** | ***p*** | **No fracture (N=2278)** | **Fracture (N=640)** | ***p*** |
| --- | --- | --- | --- | --- | --- | --- |
| Age (years) | 78.0 (75, 82) | 79 (76, 84) | <0.001 | 78 (75, 82) | 79 (76, 83) | <0.001 |
| White, % | 1715 (90.98) | 954 (93.07) | 0.05 | 2071 (90.91) | 605 (94.53) | 0.003 |
| Average wear time (days) | 5.0 (5, 5) | 5.0 (5, 5) | 0.003 | 5 (5, 5) | 5 (5, 5) | 0.54 |
| College degree or higher, % | 1479 (78.46) | 832 (81.17) | 0.08 | 1780 (78.14) | 535 (83.59) | 0.003 |
| Married, % | 1509 (80.05) | 777 (75.80) | 0.008 | 1788 (78.49) | 501 (78.28) | 0.91 |
| Chronic health conditions, % |  |  |  |  |  |  |
| 0-1 conditions | 1110 (58.89) | 505 (49.36) | <0.001 | 1273 (55.93) | 345 (53.91) | 0.64 |
| 2-3 conditions | 720 (38.20) | 457 (44.67) |  | 913 (40.11) | 267 (41.72) |  |
| >4 conditions | 55 (2.92) | 61 (5.96) |  | 90 (3.95) | 28 (4.38) |  |
| Diabetes history, % | 264 (14.01) | 169 (16.49) | 0.07 | 348 (15.28) | 87 (13.59) | 0.29 |
| Self-reported good/excellent health, % | 1686 (89.59) | 850 (82.93) | <0.001 | 1983 (87.16) | 560 (87.50) | 0.82 |
| Current smoker, % | 33 (1.75) | 19 (1.85) | 0.88 | 39 (1.71) | 14 (2.19) | 0.43 |
| Average time spent in activities of the following intensities (min/day) |  |  |  |  |  |  |
| Sedentary (<1.5 METS) | 839.2 (764.2, 906.8) | 851.8 (780.2, 929.8) | <0.001 | 844.8 (771.6, 912.6) | 843.1 (773.5, 922.4) | 0.51 |
| Light (1.5-<3.0 METS) | 65.8 (45.6, 90.0) | 59.4 (38.0, 84.2) | <0.001 | 64.0 (42.4, 88.3) | 63.1 (42.2, 89.0) | 0.91 |
| Moderate (3.0-<6.0 METS) | 68.6 (39.6, 107.4) | 58.0 (32.8, 96.2) | <0.001 | 65.5 (37.2, 104.0) | 62.6 (35.3, 104.3) | 0.61 |
| Vigorous (6.0+METS) | 4.6 (1.5, 10.6) | 3.4 (0.8, 8.6) | <0.001 | 4.0 (1.2, 9.6) | 4.6 (1.1, 10.8) | 0.22 |
| Any intensity (>1.5 METS) | 139.8 (91.2, 207.8) | 125.8 (47.7, 191.0) | <0.001 | 134.4 (84.8, 202.8) | 132.4 (79.7, 200.1) | 0.93 |
| PASE Score | 137.5 (91.7, 179.6) | 122.2 (78.2, 167.2) | <0.001 | 133.3 (88.7, 176.0) | 131.6 (83.9, 177.3) | 0.46 |
| Average sedentary bout frequency | 17.4 (14.2, 20.4) | 16.4 (12.2, 20.2) | <0.001 | 17.0 (13.6, 20.4) | 17.0 (13.4, 20.0) | 0.46 |
| Average active bout frequency | 8.0 (5.4, 11.0) | 7.4 (4.6, 10.4) | <0.001 | 7.6 (5.0, 10.8) | 7.8 (5.0, 10.6) | 0.63 |
| Height (cm) | 173.45±6.78 | 173.29±6.92 | 0.48 | 173.24±6.88 | 173.91±6.65 | 0.05 |
| Weight (kg) | 80.3 (72.5, 89.0) | 80.3 (71.6, 89.0) | 0.74 | 80.5 (72.4, 89.5) | 79.3 (71.5, 88.0) | 0.05 |
| BMI (kg/m^2^) | 26.7 (24.5, 29.2) | 26.7 (24.4, 29.3) | 0.97 | 26.8 (24.6, 29.4) | 26.3 (24.2, 28.5) | <0.001 |
| Total hip BMD (g/cm^2^) | 0.94 (0.85, 1.04) | 0.93 (0.84, 1.03) | 0.04 | 0.95 (0.86, 1.05) | 0.88 (0.80, 0.98) | <0.001 |
| IADL Impairment (>1) | 305 (16.18) | 323 (31.60) | <0.001 | 487 (21.41) | 147 (22.97) | 0.40 |
| Mini-Mental State (0-100) | 94 (91, 97) | 94 (89, 97) | <0.001 | 94 (91, 97) | 94 (90, 97) | 0.93 |
| Grip strength, kg | 40 (34, 46) | 36 (32, 44) | <0.001 | 40 (34, 46) | 38 (32, 44) | <0.001 |
| Gait speed (m/s) | 1.16 (1.03, 1.30) | 1.11 (0.92, 1.26) | <0.001 | 1.15 (0.99, 1.28) | 1.15 (0.99, 1.29) | 0.80 |
| Chair stand per 10 seconds | 4.6 (3.8, 5.4) | 4.1 (3.1, 5.0) | <0.001 | 4.5 (3.6, 5.3) | 5.2 (3.4, 5.2) | 0.001 |

**Supplementary Table 1.** Sample characteristics at the Year 7 visit for the MrOS men included in this analysis by fall status at follow-up and by incident fracture.

*N=39 participants had missing data for at least one year of follow-up, so we cannot confirm if they had recurrent falls that year. These people were categorized as non-recurrent fallers since they did not have recurrent falls during the years that data were available.

*Note.* Continuous variables presented as median (quartile 1, quartile 3) or mean±SD, and categorical variables presented as n (%). P-value assessed using Wilcoxon tests for continuous variables, and chi-squared tests for categorical variables. METS = metabolic equivalents, PASE = physical activity scale for the elderly, BMD = bone mineral density, IADL = instrumental activity of daily living.

**Supplementary Table 2**. Association between recurrent falls with frequency of breaks from sedentary bouts and active bouts after adjusting for Model 2 plus potential mechanisms linking bouts with falls using Cox proportional hazards regression models.

| Odds ratio (95% CI) | | | | |
| --- | --- | --- | --- | --- |
|  | Gait speed (m/s) | Grip strength (kg) | Chair stand (per 10 seconds) |  |
| Frequency of breaks from sedentary bouts |  |  |  |  |
| Q1 (1.4-<13.6 breaks from sedentary bouts) | 1.00 (Referent) | 1.00 (Referent) | 1.00 (Referent) |  |
| Q2 (13.6-<17.0 breaks from sedentary bouts) | 0.85 (0.69, 1.05) | 0.85 (0.69, 1.06) | 0.87 (0.70, 1.08) |  |
| Q3 (17.0-<20.4 breaks from sedentary bouts) | 0.82 (0.66, 1.03) | 0.84 (0.67, 1.05) | 0.85 (0.68, 1.06) |  |
| Q4 (20.4-34.6 breaks from sedentary bouts) | 1.06 (0.84, 1.32) | 1.03 (0.82, 1.30) | 1.09 (0.87, 1.37) |  |
| p-value^d^ | 0.02 | 0.02 | 0.03 |  |
|  |  |  |  |  |
| Frequency of active bouts per day |  |  |  |  |
| Q1 (0-<5.0 bouts) | 1.00 (Referent) | 1.00 (Referent) | 1.00 (Referent) |  |
| Q2 (5.0-<7.6 bouts) | 0.93 (0.75, 1.15) | 0.95 (0.76, 1.17) | 0.94 (0.76, 1.16) |  |
| Q3 .6-<10.8 bouts) | 0.95 (0.77, 1.18) | 1.00 (0.80, 1.24) | 0.98 (0.79, 1.22) |  |
| Q4 (10.8-26.4 bouts) | 1.05 (0.83, 1.32) | 1.07 (0.85, 1.35) | 1.10 (0.87, 1.38) |  |
| p-value^d^ | 0.03 | 0.03 | 0.03 |  |

*Note*. Frequency of breaks from sedentary bouts was defined as the number of times per day each participant had uninterrupted sedentary time lasting 5 minutes or more, excluding sleep. Frequency of active bouts were defined as the number of times per day each participant had uninterrupted active time lasting five minutes or more. Model adjusted for variables in model 2 (Age, race, clinic, season when accelerometer was worn, height, weight, self-reported history of diabetes, health status, smoking, number of comorbidities, instrumental activities of daily living impairments, total hip bone mineral density, fracture history, and fall history) plus the variable listed in the column header.

**Supplementary Table 3.** Competing risk of death with incident fracture risk in the association between recurrent falls with frequency of breaks from sedentary bouts and active bouts after adjusting for Model 2 plus potential

| Hazard Ratio (95% CI) | | |
| --- | --- | --- |
|  | Death | Incident Fracture |
| Frequency of breaks from sedentary bouts |  |  |
| Q1 (1.4-<13.6 breaks from sedentary bouts) | 1.00 (Referent) | 1.00 (Referent) |
| Q2 (13.6-<17.0 breaks from sedentary bouts) | 0.80 (0.67, 0.96) | 0.94 (0.74, 1.19) |
| Q3 (17.0-<20.4 breaks from sedentary bouts) | 0.81 (0.68, 0.96) | 1.05 (0.83, 1.32) |
| Q4 (20.4-34.6 breaks from sedentary bouts) | 0.75 (0.62, 0.92) | 0.86 (0.67, 1.11) |
|  |  |  |
| Frequency of active bouts per day |  |  |
| Q1 (0-<5.0 bouts) | 1.00 (Referent) | 1.00 (Referent) |
| Q2 (5.0-<7.6 bouts) | 0.83 (0.70, 0.98) | 0.95 (0.74, 1.21) |
| Q3 .6-<10.8 bouts) | 0.73 (0.61,0.88) | 1.04 (0.82, 1.31) |
| Q4 (10.8-26.4 bouts) | 0.67 (0.55, 0.82) | 0.97 (0.75, 1.25) |

*Note*. Frequency of breaks from sedentary bouts was defined as the number of times per day each participant had uninterrupted sedentary time lasting 5 minutes or more, excluding sleep. Frequency of active bouts were defined as the number of times per day each participant had uninterrupted active time lasting five minutes or more.

**Supplementary Figure 1.** Illustrative example of awake period from the accelerometer used for the analysis, and illustrative examples of frequency of breaks from sedentary bouts and active bouts for one hour of the day.


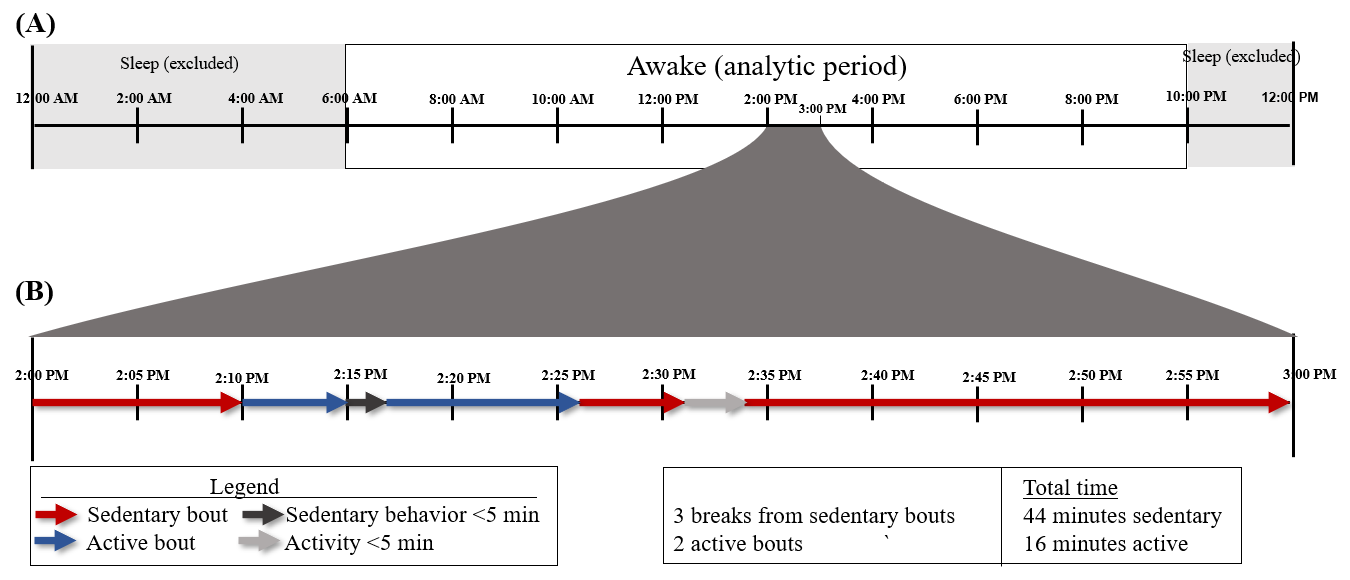


*Note*. This figure is a hypothetical example of (A) the analytic period used for time awake (sleep excluded). Sleep was determined for each person individually. The awake time of 6AM-10PM was shown here for illustrative purposes; (B) an hour of the day broken down into five-minute intervals. Red arrows represent sedentary bouts, defined as continuous sedentary behavior lasting 5+ minutes. Blue arrows represent active bouts, defined as continuous activity lasting 5+ minutes. Black arrow represents sedentary behavior that would not contribute towards the number of sedentary bouts for that day because the time sedentary did not last 5+ minutes. Grey arrow represents activity that would not contribute towards the number of active bouts for that day because the time active did not last 5+ minutes.
